# k_L_a as a predictor for probe-independent mammalian cell bioprocesses in orbitally shaken bioreactors

**DOI:** 10.1186/1753-6561-5-S8-P36

**Published:** 2011-11-22

**Authors:** Stéphanie Tissot, Dominique T Monteil, Lucia Baldi, David L Hacker, Florian M Wurm

**Affiliations:** 1 Laboratory of Cellular Biotechnology, Faculty of Life Sciences, Ecole Polytechnique Fédérale de Lausanne, 1015 Lausanne, Switzerland

## Background

Orbitally shaken flasks are commonly used at an early stage of bioprocess development with mammalian cells. In contrast to large-scale stirred-tank bioreactors, shaken flasks are usually operated in probe-independent bioprocesses, i.e. without strictly controlling the pH or dissolved oxygen concentration (DO). As a consequence, gas transfer issues are thought to limit the effectiveness of orbitally shaken flasks and bioreactors (OSRs). To define optimal operating conditions for probe-independent bioprocesses in OSRs, we tested the effects of the mass transfer coefficient of oxygen (k_L_a) on mammalian cell growth, recombinant protein production, and environmental conditions of the culture (pH, DO).

## Materials and methods

The k_L_a was measured by the dynamic method described in [1] using non-invasive O_2_ sensors (PreSens, Regensburg, Germany). A recombinant CHO DG44-derived cell line expressing a human IgG monoclonal antibody (CHO-IgG) [3] was cultivated in suspension as described [4]. To investigate the effects of the k_L_a on cell growth, CHO-IgG cells were into 1-L cylindrical bottles with working volumes from 200 to 600 mL. The bottles were equipped with vented caps and orbitally shaken at 110 rpm in an incubator at 37°C with 5% CO_2_. To test the k_L_a as a scale-up factor, CHO-IgG cells were inoculated at 0.3 million cells/mL in a 200-L OSR (Kühner AG, Birsfelden, Switzerland) with a working volume of 100 L and agitated at 57 rpm. Air containing 5% CO_2_ was flushed into the OSR at 1 L min^-1^. After overnight cultivation, samples were withdrawn from the 100-L culture and used to inoculate satellite cultures in 1- and 5-L bottles with vented caps. The volume of the cultures in bottles was adjusted to obtain the same k_L_a as the one in the 200-L bioreactor (7 h^−1^), and the bottles were agitated at 110 rpm.

## Results

**Figure 1:**
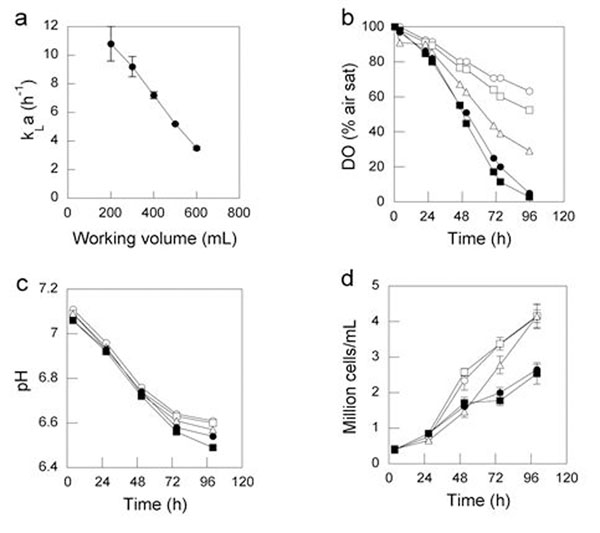
Effects of the k_L_a on CHO-IgG cell cultures. The k_L_a was measured in 1-L OSR with working volumes from 200 to 600 mL (a). The CHO-IgG cells were cultivated in 1-L OSR in 200 (○), 300 (□), 400 (∆), 500 (●) and 600 mL (■). The DO (b), pH (c) and viable cell density (d) were measured at the times indicated. The shaking diameter was 5 cm.

In a 1-L OSR the k_L_a decreased from 11 to 3 h-1 as the working volume increased from 200 to 600 mL (Fig. 1a). As the working volume of the cultures increased in the 1-L OSR, the DO decreased (Fig. 1b). In all the cultures, the pH decreased with time of cultivation (Fig. 1c) At working volumes greater than 400 mL (k_L_a <7 h^−1^), the maximal cell density was about 40% less than in cultures of ≤ 400 mL (Fig. 1d).

To test the k_L_a as a scale-up factor for probe-independent bioprocesses, CHO-IgG were inoculated in a 200-L OSR. After overnight incubation, samples of the 100-L culture were used to inoculate satellite cultures in 1- and 5-L OSRs at volumes to give k_L_a values of 7 h^-1^. The cell densities were similar in the 1-, 5- and 200-L OSRs and reached 3.5 million cells/mL after 90 h (data not shown). The recombinant IgG concentrations at this time were about 150 mg/L. The pH decreased from 7.25 to 6.7 in all the cultures (data not shown), and the glucose, glutamine, lactate and glutamate profiles were similar in all the cultures.

## Conclusions

Our results indicate that the k_L_a is a good parameter to predict suitable conditions for cell cultures in probe-independent OSRs. Furthermore, our study demonstrates that cultures having different nominal scales but the same k_L_a also had the same cell growth, recombinant protein production, and culture conditions (pH and DO). The minimal k_L_a required to avoid pH and DO limitations in OSRs was 7 h^-1^ for CHO-IgG cells. Cell cultivation in a 200-L OSR without pH or DO controllers resulted in similar cell densities, recombinant protein titers and pH values as in 1- and 5-L OSRs when the three types of OSRs were operated at the same k_L_a. These results suggest that large-scale bioprocesses can be operated without pH or DO controllers as long as a sufficient k_L_a is maintained through appropriate cultivation conditions (e.g. working volume, agitation rate, geometry of the vessel).
